# Use of Herbal Medicine Is Associated With Late-Stage Presentation in Tanzanian Patients With Cancer: A Survey to Assess the Utilization of and Reasons for the Use of Herbal Medicine

**DOI:** 10.1200/GO.22.00069

**Published:** 2022-07-12

**Authors:** Oliver Henke, Walter Bruchhausen, Anna Massawe

**Affiliations:** ^1^ Cancer Care Centre, Kilimanjaro Christian Medical Centre, Moshi, Tanzania; ^2^ Section Global Health, Institute for Hygiene and Public Health, University Hospital of Bonn, Bonn, Germany

## Abstract

**PURPOSE:**

In Tanzania, patients with cancer present late with advanced stages. Among other reasons, the use of herbal medicine (HM) might contribute to delay in diagnosis and treatment. This study aims to understand the utilization of HM and the reasons for its' use.

**METHODS:**

A semistructured 30-item questionnaire with closed- and open-ended questions was applied to a cohort of patients with cancer at Kilimanjaro Christian Medical Centre. Content analysis was performed for answers to open-ended questions.

**RESULTS:**

Three hundred two patients were interviewed, 50.9% males and 49.1% females. The mean age was 64 years. 71.6% were in stages III/IV. 70.5% patients used HM and 67.1% started conventional medicine after stopping HM, 7.5% started HM after conventional medicine, and 24.9% combined both. Stage IV patients used HM as first line significantly more often in comparison: stage I 55.6%; II 58.1%; III 57.2%; and IV 70.6%. 77.5% did not tell their doctors about the use of HM. Commonest reasons to use HM were belief in cure, hope of symptom relief, lack of cancer services, and tradition.

**CONCLUSION:**

The majority of patients used HM before consulting a doctor, which contributes to stage IV presentation. Use of HM alongside with chemotherapy is mostly not known to the treating physician and may lead to interactions. These findings must alert health care workers and health policymakers to further foster health education about cancer and its treatment. Further research is needed to understand the wide use of HM among Tanzanian patients with cancer and the role that traditional and spiritual healers play in the field of cancer care.

## BACKGROUND

Cancer diseases are a growing burden in Tanzania and sub-Saharan Africa (SSA) as a whole^1^ and will continue to affect larger swathes of the population in the future. Tanzania had a prevalence of 73,303 cancer cases in 2020 with an annual incidence of 40,464 and 26,945 cancer-related deaths.^2^ Three cancer treatment facilities exist in the country with a population of almost 60 million, and barriers to access treatment in this setting include long distances to receive diagnosis and treatment, high out-of-pocket expenditures, and low level of knowledge among patients and health care providers about cancer.^3,4^

**CONTEXT**

**Key Objective**
Why do Tanzanian patients with cancer use herbal medicine (HM) and does the use influence time to presentation?
**Knowledge Generated**
Our survey revealed that 70% of patients with cancer use HM for their current cancer disease because they believe in cure through HM, expect symptoms relief, and because of lacking cancer services in their proximity. Most patients use HM before seeking care at a hospital, which leads to significant higher proportion presenting in stage IV disease compared with those who first consulted a conventional health facility (*P* = .0013).
**Relevance**
Our survey proofed the assumption that the use of alternative medicine leads to late-stage presentation in cancer diseases. The high proportion of patients using HM before seeking care at a hospital is alarming and reflects the deep-rooted tradition of HM. Efforts must be taken to educate patients on the relevance of timely presentation and treatment initiation of cancer diseases.

Apart from these impediments to receive medical attention in low- and-middle-income countries, it is known that patients with cancer worldwide use traditional and herbal medicine (HM) generally on a large scale^5^ and in SSA, this is a critical component of health care.^6^ The use of traditional and HM among patients with cancer in SSA has been studied in a few countries only and display inhomogeneous findings, from Nigeria, where 34% of patients with cancer use it, to 74% in Ethiopia.^7^ As most of the patients use alternative medicine before consulting a medical doctor (respectively a conventional health care facility),^8^ it is likely that it contributes to the high number of late-stage presentation of patients with cancer in SSA.^3,9,10^ But, data confirming this assumption are lacking.

We conducted this study to understand the utilization of HM among patients with cancer attending the Cancer Care Centre (CCC) at Kilimanjaro Christian Medical Centre (KCMC) and the reasons behind their choice to identify possible interference with early presentation of cancer diseases and treatment. A better understanding of the use of HM in Tanzania should also serve to shape health education and individual counseling of patients.

## METHODS

Participants have been recruited between May and July 2018. A convenient sampling of patients with cancer visiting the CCC at KCMC for either clinic or treatment appointments have been invited to participate.

KCMC is the referral and university hospital in Northern Tanzania, located in the city of Moshi, and serves a catchment area of approximately 12 million people, reaching into neighboring Kenya. The CCC was established in 2016 and is at present the third cancer treatment facility in the country and the only one in the northern zone of the country.

A semistructured 30-item questionnaire with closed-ended, binary, multiple choice, and open-ended questions was applied. Seven items documented sociodemographic variables (age, sex, occupation, marital status, level of education, district of residency, and tribe), six items were directed to the current cancer disease and treatment, and 17 items were directed to the use of HM (Table 1). The Swahili term used to ask for HM was *dawa ya mitishamba*, which is equivalent to *HM or medicinal plants* and describes fresh plant products as well as processed powder from HMs. The survey was conducted by a trained and specialized nurse in palliative care, and a prephase with five patients secured the applicability of the questionnaire. The interviews were conducted in a separate room at CCC to secure privacy.

**TABLE 1:**
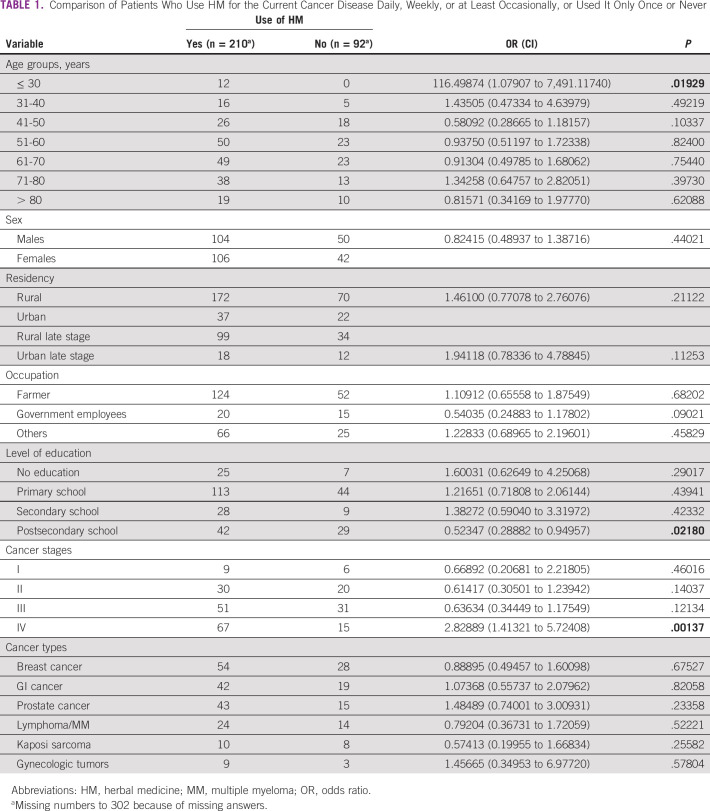
Comparison of Patients Who Use HM for the Current Cancer Disease Daily, Weekly, or at Least Occasionally, or Used It Only Once or Never

Answers to closed-ended and multiple choice questions have been coded, and bivariate analyses have been performed where indicated. A content analysis of the open-ended questions (reasons to use HM) has been performed and categories developed.

Ethical approval was sought and granted by the Ethical Review Board of the Kilimanjaro Christian Medical University College. A consent form has been signed by all participants. The participants were equipped with a telephone number of the study nurse in case of further questions or seeking advice or help after the interviews. We conducted the study in accordance with the Helsinki Declaration of the World Medical Association.

## RESULTS

In total, 302 participants were interviewed; sex was balanced, with 154 (50.9%) males and 148 (49.1%) females. The average and mean age was 64 years, and 59 (19.5%) lived in urban and 242 (80.1%; one missing) in rural areas. The majority of participants were farmers (176; 58.3%), and teachers and other government employees (35; 11.6%). Two hundred four participants were married (67.6%), 49 were widowers (16.2%), 33 were single (10.9%), and 16 (5.3%) were divorced.

The level of education ranged from no education (32; 10.6%), primary school only (157; 51.9%), secondary education (37; 12.3%), to postsecondary education (71; 23.5%; five missing).

The most common type of cancer was breast cancer (82; 27.2%), GI cancer (61; 20.2%), prostate cancer (58; 19.2%), lymphoma (including multiple myeloma; 38; 12.6%), Kaposi sarcoma (18; 5.9%), gynecologic cancer (12; 3.9%), and other entities (32; 10.6%).

Fifteen patients (4.9%) were in stage I at the time of interview, 50 (16.6%) in stage II, and 82 patients in the stages III and IV (27.2%). Seventy-three patients did not know their stage (24.2%).

**FIG 1:**
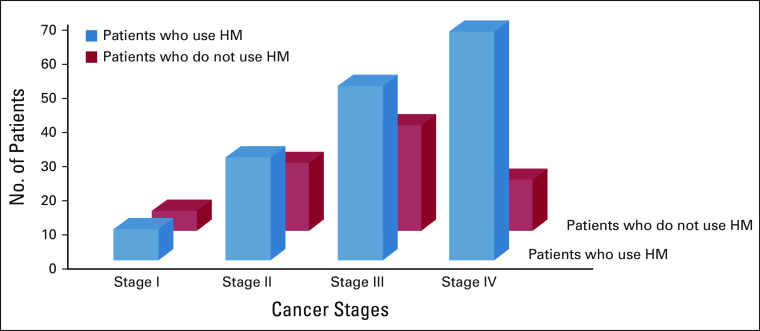
Use of HM according to disease stages. HM, herbal medicine.

Two hundred eleven patients have ever used HM (69.9%) before being diagnosed with the current cancer disease. Two hundred thirteen patients use HM in the current disease: 193 (63.9%) daily, eight (2.6%) weekly, nine (3.0%) occasionally, and three patients (1.0%) only one time (Fig 1).

In addition to the use of HM, 77 patients (25.5%) consulted a traditional healer for the current cancer disease. Out of those 77, 20 (26.0%) patients have visited the traditional healer once (26.0%), 22 (28.6%) twice, and 35 (45.5%) several times.

The majority of patients who used traditional medicine in the current cancer disease (n = 213) have started conventional medicine (CM) after stopping HM (143 [67.1%]), 16 started HM after finalizing conventional treatment (16 [7.5%]), and 53 patients (24.9%) combined HM and conventional treatment. Stratified to cancer stages, patients in stage IV have used HM before CM in 70.6%, HM/CM at the same time in 27.9%, and CM before HM in 1.5%. Compared with the other stages (stage I: 55.6%; 11.1%; 33.3%; stage II: 58.1%; 9.7%; 32.3; stage III: 57.2%; 24.6%; 17.7%), this difference is significant with an odds ratio of 12.000 (CI, 1.53303 to 258.38659; *P* = .00383).

One hundred fifty-seven (73.7%) out of the 213 patients using HM for the current cancer disease reported about a poor level of satisfaction with HM, 52 (24.4%) report a good satisfaction, and three (1.4%) patients had excellent satisfaction. Corresponding to the level of satisfaction, 157 (73.7%) patients would not use HM for a cancer disease again nor recommend HM for cancer diseases but 55 (25.8%) would do.

One hundred sixty-five patients (77.5%) did not tell their oncologists about the use of HM.

One hundred five patients got their information about HM from family members, 131 from friends, 78 through media, 42 from other patients, and 35 from health care workers.

**FIG 2:**
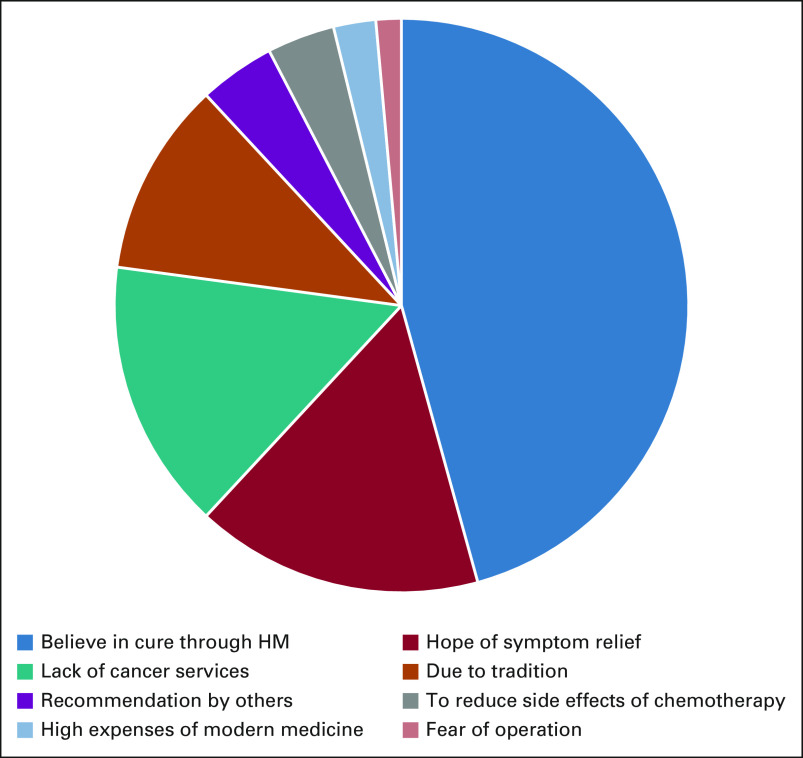
Reasons of the interviewed patients for the use of HM. HM, herbal medicine.

The answers were categorized after content analysis into eight categories: believe in cancer cure through HM (mentioned by n = 96), hope of symptom relief (n = 34), lack of cancer services in their area (n = 32), because of tradition (n = 23), recommendations by others (n = 9), to reduce the side effects of chemotherapy (n = 8), high expenses (n = 5), and fear of operation (n = 3; Fig 2).

## DISCUSSION

This survey was conducted to understand the use of HM among patients with cancer in Northern Tanzania. Almost two third of the patients indicated to have used or still use HM for the current cancer disease that has let them to attend the cancer care clinic. Hope for cure and tradition have been mentioned as the most common reasons to use HM followed by lack of cancer services.

An unexpected finding in our cohort was the finding of high utilization of HM among patients younger than 30 years, which stands in stark contrast to a household survey from South Africa in 2011, where age was adversely associated with the use of traditional healers.^11^ Although our results must be taken with caution because of the few numbers (n = 12) of interviewees in this group, the fact that all these patients indicated the use of HM deserves some thoughts and discussion. Sayed et al^12^ examined young Kenyans (median age 22 years in women and 31 years in men) by means of focus group discussions and questionnaires. Although the indication of the use of traditional healers was generally very low in the questionnaires, the focus group decision revealed insights into believes of being bewitched, especially in not common diseases such as cancer.^12^ Another important factor for the use of alternative medicine was the proximity of healers and hence their easy access.

It must be noted from our findings that 70% used HM for the current cancer disease but only a quarter of the patients consulted a traditional healer. Hence, using HM must be influenced by other factors. Hereby, friends and media as sources of information have been named by the interviewees in our cohort. Corresponding to this, Asuzu^13^ stated in a 1994 published article from Ibadan in Nigeria that the most common source of information about HIV are media. In a recent publication from South Africa about health promotion and social media, a major challenge mentioned was “it allows information to spread far and fast […] irrespective of the source of information.”^14^ We do believe that the increasing and constant availability of social media information about health and medicine has a huge impact especially on younger patients and might explain our findings. Traditional medical practitioners use media on a large scale to advertise their products and do this in a manner that lacks both medical and business ethics, according to a study analyzed the situation in South Africa, Mozambique, and Zimbabwe.^15^ This, however, remains an assumption, and further research is needed to look closer to the relation of social media influence on patients with cancer.

**FIG 3:**
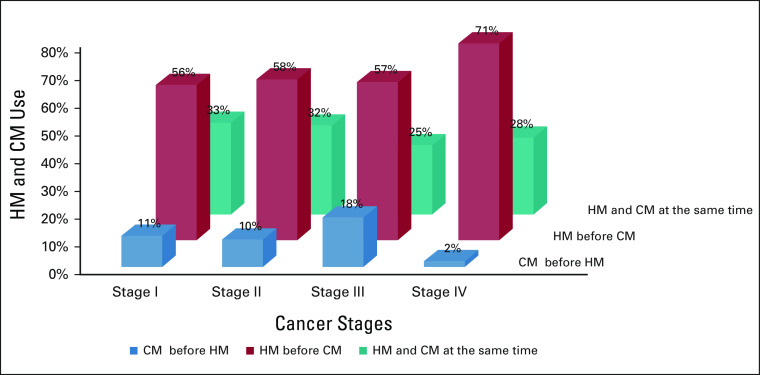
Sequence of using HM and CM according to stage of cancer disease. CM, conventional medicine; HM, herbal medicine.

The correlation of stage IV cancer disease and use of HM is—apart from the younger age group—the only significant finding of our survey. Facing late-stage cancer diseases, patients often seek alternative treatment, often at the same time.^16-18^ However, our findings show that more than 70% use HM before seeking medical attention in the CCC and only 2% started with CM before using HM (Fig 3). The authors understand this as a strong but also alarming evidence that HM distracts patients for a long time from being served in modern health facilities. This finding is supported by other studies from different countries.^9,19,20^

The scope of reasons given for the use of HM covers the whole range of motives and obstacles for seeking any form of therapy, from basic mental issues such as trust, habit, fear, or social pressure, to practical issues such as distances and finances. The Human Right to Health addresses most of these concerns as requirements by the AAAQ approach (Availability, Accessibility, Acceptability, and Quality), that is, that health care services must be available and accessible, including being affordable and culturally acceptable, and of sufficient quality.^21^

Although only a small number of our interviewees expressed that high expenses of modern medicine was a reason for using HM, it must be assumed that it is a considerable factor for treatment decisions, especially when the treatment goes beyond primary care. The studies mentioned in the introduction proved this point.^3,4^ It has been also shown that an increase in treatment costs induces patients who had been open to biomedical services to return to traditional medicine.^22^ The fact that many cancer medicines have been provided free of charge in the Centre during 2018 through donations might have led to mitigate the financial problem in our cohort.

The fact that patients from rural areas are more likely (odds ratio 1.46) to use HM than those from urban areas indicates the importance of availability and geographical accessibility of treatment in specialized centers.^10^

By far, the highest share in the survey has been the socially expected answers of sociocultural factors of own belief, recommendation, and tradition. Use of HM appears as the common way of life. Yet, HM is not a traditional, that is, ancient complex of fixed practices for dealing with unwanted states of health, but was formed in competition, imitation, and resistance with regard to the practices imported from the Global North.^23^ Thus, it is not a mere matter of just being accustomed to traditional medicine but involves questions of constructing cultural identity and securing social cohesion, dealing with conflict, and coping with contingency. The majority of these concerns are not antimodern or anti-Western, but deeply connected to the ways of explaining misfortune, defending oneself against destructive forces. As CM does not address these existential issues, regarded as the ultimate causes of ill-health, a complementary action must be applied. These beliefs are expressed by the high number of interviewees stating that only HM can cure cancer. Furthermore, many patients started using both types of medicine simultaneously. This may also point out to the conviction that conventional cancer treatment cannot be sufficient for getting completely healed. And furthermore, some patients mentioned the use of HM to reduce the side effects of chemotherapy treatment. This is also known as a common motivation for patients with cancer in different settings.^24-26^

The majority of the interviewed patients would not talk to their oncologists about the concurrent use of HM. This correlates with findings from other parts of the world, such as Korea, where Kang et al^27^ revealed that 70% of the patients with cancer would not talk with the treating physician about the use and a Malaysian study with self-administered questionnaires displayed similar results,^28^ as did studies conducted in African countries.^25,26^ Patients would not relate this information to their doctors because they were not asked for it. Fear of telling the physician or the belief that HM has no side effects of interaction is also common in patients with cancer.^29^

In conclusion, the use of HM is generally high among patients with cancer in Northern Tanzania and most use it before consulting a medical doctor, which contributes to delay in diagnosing and eventually treatment of the cancer disease. The concurrent use of HM alongside chemotherapy is mostly not known to the treating physician and may lead to unwanted interactions. This finding must alert Tanzanian oncologists (and globally as other studies have shown) to thoroughly obtain the history of patients including the pointed question about HM use and the reasons behind it.

Misconceptions about conventional cancer treatments must be responded by enhanced health education of the general population as no specific correlation with demographic characteristics can be identified, neither in our study nor in most of others.

This survey was physically conducted within the CCC's building. A bias toward social desirable answers is likely, which might indicate an even higher utilization of HM.

Furthermore, our study cannot distinguish within the highly diverse practices of traditional medicine, for example, whether the herbal treatment was combined with other rituals or what the role of traditional and spiritual healers has been in the decision for the use of HM. Future more qualitative studies should apply such differentiations to understand what precisely patients with cancer are looking for. This might also facilitate a dialogue with healers who provide HMs about referral and possible collaboration as well as a culturally sensitive health education and promotion for the general population. With the rising burden of noncommunicable diseases, such approaches may increase the urgently needed acceptance of timely and long-term treatment for health problems that start less obviously than many infectious diseases.

## Data Availability

The data that support the findings of this study are available from the corresponding author upon reasonable request.
